# Neonatal Perforated Appendicitis

**Published:** 2012-01-01

**Authors:** Kanchan Kayastha

**Affiliations:** Department of Paediatric Surgery, The Children's Hospital and the Institute of Child Health Lahore, Pakistan

**Dear Sir,**

Appendicitis is the most common cause of acute surgical abdomen in children and adults but very rare in infants and neonates. Nonspecific clinical features and low index of suspicion make its diagnosis and management challenging which may result in high chances of complications like perforation and peritonitis thus increasing the morbidity and mortality. Appendicular perforation leading to peritonitis is a life threatening condition in neonates [1-3]. We are presenting a case of neonatal appendicular perforation leading to peritonitis.

A 12-day-old male baby weighing 3kg was admitted to neonatal emergency with progressive abdominal distension, fever, irritability, and reluctance to feed. Baby was well for 10 days when he developed the symp­toms. On examination the baby was febrile (101F), tachycardia (150/min), mild respiratory distress and abdominal distension with generalized tenderness. Patient was dehydrated and irritable. Immediate first line management was given. Complete blood picture revealed leococytosis. X ray abdomen showed pneumoperitonium. Patient was stabilized and operated.

At laparotomy, marked amount of gas present in abdominal cavity. Bowel loops were adherent with inflammatory flakes. On further exploration perforated appendix was found (Fig. 1). The base of the appendix was perforated causing peritonitis. There were no necrotic patches on the gut. Large gut was of normal caliber. Appendectomy was done and rectal biopsy was taken. Abdomen washed with warm saline and closed in layers. The post operative recovery was uneventful. The biopsy report excluded hirschsprung’s disease where as appendix revealed acute inflammatory changes with lymphatic hyperplasia.

**Figure:**
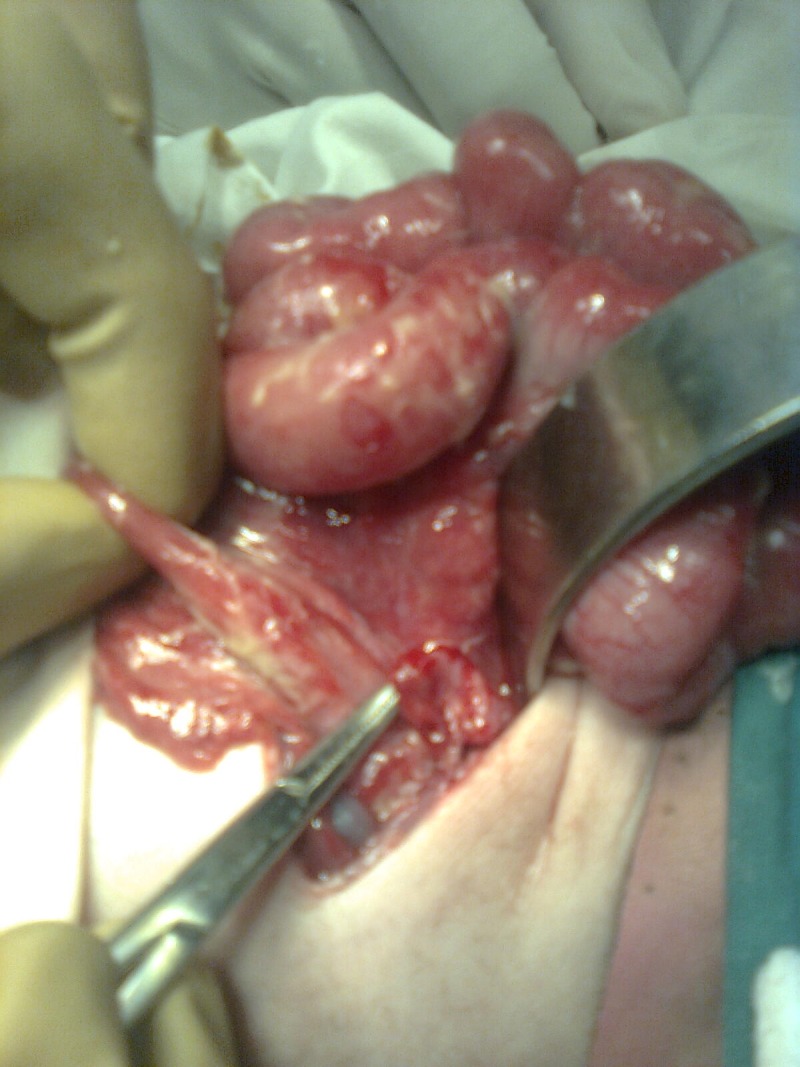
Figure 1: Perforated base of appendix

Acute appendicitis is a rare cause of acute abdomen in neonates and never considered in the differential diagnoses. The funnel shaped wide base of appendix, liquid diet and recumbent position of the neonates are less likely to cause acute appendicitis. It is extremely difficult to make a diagnosis of acute appendicitis in neonates due to non specific signs and symptoms that usually result in perforation of the appendix. The appendicular perforation can lead to pneumoperitonium as in the index case.

Perforation could be due to sole inflammation of appendix or could be the end result of another disease processes such as hirschsprung’s disease, necrotizing enterocolitis, cystic fibrosis etc. A thin appendicular wall and indistensible cecum predisposes to appendicular perforation in neonatal appendicitis. The treatment of perforated appendicitis in neonates is appendectomy, however, where there is strong suspicion of hirschsprung’s disease on account of delayed passage of meconium or operative findings suggest it a colostomy may be performed with serial biopsies. In absence of suspicion of hirschsprung’s disease simple appendectomy with peritoneal lavage with warm saline is indicated; rectal biopsy may be added to exclude hirschsprung’s disease.

## Footnotes

**Source of Support:** Nil

**Conflict of Interest:** None declared

